# The mouth as a window: A multi-center retrospective study of oral extraintestinal manifestations of IBD and their management

**DOI:** 10.4317/medoral.27830

**Published:** 2025-11-22

**Authors:** Miguel A Aristizabal-Torres, Marketa Janovska, Lauren P Loeb, Yaohua Ma, Roy S Rogers-III, Francis A Farraye, Alison J Bruce, Victor Chedid, Jana G Hashash, Katherine J Bodiford

**Affiliations:** 1 Department of Dermatology, Mayo Clinic, Jacksonville, Florida, USA; 2 Institute of Dental Medicine, First Faculty of Medicine and General University Hospital, Charles University, Prague, Czech Republic; 3 Department of Internal Medicine, Mayo Clinic, Jacksonville, Florida, USA; 4 Division of Clinical Trials and Biostatistics, Mayo Clinic, Jacksonville, Florida, USA; 5 Department of Dermatology, Mayo Clinic, Scottsdale, Arizona, USA; 6 Division of Gastroenterology and Hepatology, Mayo Clinic, Jacksonville, Florida, USA; 7 Division of Gastroenterology and Hepatology, Mayo Clinic, Rochester, Minnesota, USA

## Abstract

**Background:**

Up to 50% of patients with inflammatory bowel disease (IBD) have associated oral extraintestinal manifestations (OEIMs). We aim to describe the prevalence of OEIMs in IBD and propose a therapeutic algorithm.

**Material and Methods:**

Electronic health records of adult patients with IBD who presented with at least one oral symptom between January 2017 and November 2021 at a tri-state tertiary academic medical center were retrospectively reviewed. Data included demographics, IBD history, oral diagnoses, OEIM treatments, clinical outcomes, and comorbidities.

**Results:**

A total of 116 patients were included; 67 (57.8%) had Crohn's disease (CD) and 49 (42.2%) had ulcerative colitis (UC). Aphthous ulcers were the most common OEIM (80.2%). Frequently used treatments included compounded or mixed mouthwashes (51.7%), topical corticosteroids (33.6%), systemic corticosteroids (20.7%), and topical anesthetics (19.8%). In CD, colchicine was significantly associated with OEIM improvement (p=0.009). In UC, systemic corticosteroids (p=0.03), colchicine (p=0.048), and topical tacrolimus (p=0.048) were significantly associated with improvement.

**Conclusions:**

OEIMs are common in IBD and can influence treatment decisions. Colchicine and topical agents demonstrated benefit in selected cases. These findings support multidisciplinary care and inform a therapeutic algorithm for OEIM management in IBD.

## Introduction

Inflammatory bowel disease (IBD) is a significant and growing public health concern worldwide. In the United States, the sex-standardized incidence of IBD is 10.9 per 100,000 person-years, and the prevalence is 721 per 100,000 population, with an estimated 2.39 million Americans diagnosed, reflecting similar trends globally. (1 , 2) IBD comprises Crohn's disease (CD) and ulcerative colitis (UC), both chronic, relapsing conditions with variable symptomatology depending on the site of gastrointestinal involvement. Patients with IBD may develop extraintestinal manifestations, including oral extraintestinal manifestations (OEIMs), which can negatively impact nutritional status and quality of life. (3) Epidemiologically, OEIMs are estimated to affect approximately 8%-50% of patients with IBD, (4 , 5) although the true prevalence is likely underreported due to the underutilization of both routine oral examinations and oral biopsies in clinical practice. (7) Despite their clinical relevance, OEIMs remain poorly characterized in the literature and represent a diagnostic and management challenge in routine care.

CD affects the gastrointestinal tract anywhere from the mouth to the anus, while UC is generally limited to the colon. Both conditions have been associated with distinct oral findings. (8) In CD, OEIMs may include indurated mucosal tags, mucosal cobblestoning, mucogingivitis, deep linear ulcerations, and orofacial granulomatosis. (9 , 10) In contrast, patients with UC have been reported to have an increased risk of developing pyostomatitis vegetans, a rare but highly specific oral manifestation. (11 , 12) In addition to these disease-specific findings, several nonspecific OEIMs have been described in both forms of IBD, including recurrent aphthous stomatitis, angular cheilitis, mucositis, atrophic glossitis, xerostomia, burning mouth syndrome, oral candidiasis, halitosis, dysgeusia, gingivitis, and periodontitis. (10 , 13 , 14) Some of these conditions may represent direct mucosal involvement related to IBD pathophysiology (14), while others may result from secondary consequences such as nutritional deficiencies or systemic inflammation. (14 - 16) Furthermore, IBD-therapies may be associated with oral disease. (10 , 15 , 17)

Oral manifestations may precede or accompany the diagnosis of IBD, and even have been postulated as potential surrogates of disease severity, making them valuable indicators for early detection and intervention. (18 , 19) Therefore, recognizing these manifestations is crucial for timely diagnosis and treatment. Herein, we report a cohort of patients with OEIMs in the context of IBD. Our findings aim to help clinicians address this underrecognized domain and ultimately optimize patient care.

## Material and Methods

As previously reported in this cohort,(19) we conducted a retrospective review of adult patients with CD or UC evaluated at Mayo Clinic campuses in Rochester, MN; Jacksonville, FL; and Scottsdale, AZ between 1 Jan 2017 and 29 Nov 2021. The present analysis is distinct in its focus on OEIMs, incorporating detailed treatment patterns and therapeutic outcomes that were not examined in the prior report.

Patients were identified via the EHR based on a confirmed diagnosis of CD or UC and 1 of the following oral diagnostic codes: K12.0 (recurrent aphthous stomatitis), K13.4 (granuloma/granuloma-like lesions of oral mucosa), K12.30 (mucositis [ulcerative], unspecified), K12.1 (other forms of oral stomatitis), or G51.2 (Melkersson's syndrome). Each record was manually reviewed to verify eligibility. All coded oral diagnoses meeting these criteria were included, even if the etiology may be secondary to IBD therapy (e.g., corticosteroids, immunosuppressants) or nutritional deficiencies. This approach was chosen to capture the full spectrum of oral pathology observed in patients with IBD. Exclusion criteria included lack of confirmed IBD in clinical documentation or absence of oral manifestations in clinical notes.

Data abstracted from the EHR included demographics, IBD history, comorbidities, oral symptoms and diagnoses, and treatments for OEIMs. Clinical notes were reviewed to determine whether clinicians considered the oral findings to be IBD-related and whether IBD was active at the time of OEIM presentation. Endoscopy reports and relevant labs were reviewed to assess IBD activity.

This study was approved by the Mayo Clinic Institutional Review Board (IRB# 21-010546). Only patients who provided authorization for the use of their health records for research purposes were included. The reporting of this study adheres to the Strengthening the Reporting of Observational Studies in Epidemiology (STROBE) guidelines.

Statistical Analysis

We used the Wilcoxon rank sum test and the Kruskal-Wallis test for continuous measures. Because of the sample size, we used the Fisher exact test for comparing categorical measures. Numeric variables were summarized with median and range, and categorical variables were summarized with frequency and percentage. All tests were 2 sided, and pvalues less than .05 were considered statistically significant. No imputations were used for missing data. Study data were analyzed with Posit software. Statistical analysis was performed using R statistical software (version 4.2.2; R Foundation for Statistical Computing).

Treatments were summarized and analyzed for their association with OEIM improvement, with analyses conducted separately for CD and UC. To assess whether the proportion of each treatment differed significantly among the five outcome groups (Improvement, Improvement with IBD treatment, Required additional therapy, Did not improve, and Outcome not documented), Fisher exact tests were performed within each disease type.

## Results

Among the previously described cohort, (19) we identified 116 patients meeting inclusion criteria for analysis (CD, n=67; UC, n=49). Demographic characteristics are presented in Table 1 and oral diagnoses are summarized in Table 2.

Table 1

Table 2

The most common oral diagnoses were aphthous ulcer (CD, 85.1%; UC, 73.5%), unspecified stomatitis (CD,11.9%; UC,14.3%), and unspecified mucositis (CD, 7.5%; UC, 12.2%). Angular cheilitis and oral candidiasis were also frequently observed in patients with CD (9% each). Other diagnoses occurred less frequently. Twenty-five patients (21.6%) had an oral biopsy performed (Table 3). Among patients with CD (n=14), findings were nonspecific for 8 (57%), although findings consistent with oral CD were observed for 3 patients (21%). Among patients with UC (n=11), findings were nonspecific for 5 patients (45%), and lichenoid inflammation was noted for 4 patients (36%).

Table 3

We previously reported data regarding the temporal relationship between oral and intestinal symptoms in this cohort. (19) A substantial number of patients had oral findings deemed to be associated with their underlying IBD (CD, n=38; UC, n=17). In many cases, OEIM activity paralleled IBD flares (CD, n=30; UC, n=17). Across the cohort, the presence of OEIMs prompted changes in IBD management, including dose adjustments of existing therapies (n=25) and the initiation of new systemic agents (n=10). (19)

Table 4 outlines specific treatments administered for oral manifestations. The most commonly used therapies included compounded or mixed mouthwashes (51.7%), topical corticosteroids (33.6%), systemic corticosteroids (20.7%), topical anesthetics (19.8%), and topical antifungals (14.7%).

Treatment responses are detailed in Table 5. Among patients with Crohn's disease, colchicine emerged as a significantly effective therapy for oral extraintestinal manifestations (p=.009). In the ulcerative colitis group, improvement in OEIMs was significantly associated with systemic corticosteroids (p=.03), colchicine (p=.048), and topical tacrolimus (p=.048).

Table 4

Table 5

As we previously reported, comorbid medical conditions were common in this cohort. Anemia was the most prevalent (50.9%), followed by nutritional deficiencies (37.1%), anxiety (36.2%), inflammatory arthritis (34.5%), and depression (32.8%), highlighting the broad systemic impact of IBD beyond the gastrointestinal tract. (19)

## Discussion

In this study, we conducted a comprehensive evaluation of OEIMs in patients with IBD with a focus on treatment of OEIMs. Several inflammatory conditions were frequently observed in both CD and UC, reinforcing the potential for OEIMs as a hallmark of IBD-associated oral pathology. These findings highlight the importance of routine oral health assessments in patients with IBD, particularly given the accessibility of the oral cavity as a potential surrogate marker for intestinal inflammation and the possible diagnostic and therapeutic relevance of OEIMs.

Aphthous ulcers were the most prevalent OEIMs, affecting over 85% of patients with CD and 73.5% with UC. This finding is consistent with previous literature suggesting a strong association between aphthous stomatitis and IBD and may reflect either a direct manifestation of IBD or a consequence of nutritional deficiencies, particularly hematinic deficiency. (4) Interestingly, while angular cheilitis and oral candidiasis were more frequently observed in CD, unspecified stomatitis and mucositis were relatively similar in both groups. These differences may reflect underlying pathophysiological distinctions between CD and UC or may be influenced by differences in treatment regimen or nutritional status. (10)

Oral candidiasis, although not traditionally classified as a primary OEIM, was observed in a notable proportion of patients with CD. Its occurrence in IBD is often secondary to corticosteroid or immunosuppressive therapy, or a consequence of malnutrition, rather than a direct immune-mediated process. We included it to reflect the breadth of oral conditions encountered in this population and to facilitate comparison with other published cohorts.

The exact mechanism underlying the development of OEIMs in IBD remains unclear. However, immune dysregulation involving cross-reactive antigens between the intestinal and oral mucosa may contribute to the mucocutaneous manifestations of IBD. (14) The proinflammatory cytokine milieu associated with IBD has also been detected in the saliva of affected individuals, with elevated levels of interleukin (IL)-1 reported in both CD and UC. (20) Additionally, increased concentrations of IL-6, IL-8, monocyte chemoattractant protein-1 (MCP-1), and tumor necrosis factor (TNF)- have been observed in patients with CD and UC. (20) Furthermore, in patients with periodontitis, bacterial overgrowth may stimulate immune cells to overexpress inflammatory mediators, contributing to a proinflammatory oral environment. (21) Consequently, OEIMs in IBD may result from elevated inflammatory cytokines in the context of immune dysregulation.

Malnutrition is a well-documented complication of IBD. (22) In our cohort, up to 50% of patients had anemia and 37% had a documented nutritional deficiency. (19) In the literature, hospitalized individuals with IBD have been found to be 2.9 to 3.1 times more likely to have protein-calorie malnutrition compared with non-IBD patients. (23) The prevalence of malnutrition among hospitalized patients has been estimated at 6.1% in those with CD and 7.2% in those with UC. (24) OEIMs in patients with IBD and malnutrition are varied and may include atrophic glossitis, angular cheilitis, and recurrent aphthous stomatitis, among others. (10) Dental conditions, including gingivitis, periodontitis, and dental caries, may be more prevalent in patients with vitamin D deficiency and could contribute to the overall inflammatory burden observed in inflammatory bowel disease. (25) In selected patients, symptoms such as burning sensation and altered taste perception may also be present. (10) The presence of OEIMs in patients already at risk for malnutrition may further compromise their nutritional status, and we believe this warrants comprehensive evaluation.

Biopsies were performed in approximately one-fifth of the cohort, with most histopathologic findings being nonspecific. 21% of CD patients had biopsy features consistent with oral CD, while lichenoid inflammation was observed in a notable proportion (36%) of UC patients. These findings highlight the limited diagnostic specificity of oral biopsies in the context of IBD, suggesting that clinical correlation remains essential for the diagnosis and management of OEIMs.

The prevalence of nonspecific histologic findings raises questions about other potential contributors to OEIM pathogenesis beyond immune dysregulation and malnutrition, namely, dysbiosis. (26) The gastrointestinal microbiota in patients with IBD differs significantly from that of individuals without IBD and is considered part of the disease's multifactorial etiology. A correlation has been identified between specific bacterial overgrowth and elevated levels of IL-1 and lysozyme, suggesting that dysbiosis may play a role in the underlying pathogenic mechanisms. (20) Furthermore, the saliva of patients with IBD has been shown to harbor distinct bacterial populations associated with dysbiosis, which are linked to a pronounced proinflammatory oral microenvironment. (14) This microbial imbalance may further promote immune dysregulation, potentially leading to secondary inflammation and the development of OEIMs.

Although infrequent, therapies used in IBD may be associated with the development of OEIMs, either through immunosuppressive effects or direct mucosal toxicity. (10) Beyond the classical concern of increased susceptibility to oral opportunistic infections with systemic immunosuppressants, certain medications have been linked to specific oral manifestations. Aminosalicylates and biologics, although rarely, have been associated with the development of oral lichen planus or lichenoid drug reactions. (15 , 17) The risk of pulp obliteration and calcification related to corticosteroid use, particularly in children, should not be overlooked. (27) Methotrexate has been implicated in the development of aphthous ulcers, ulcerative stomatitis, and mucositis. (28)

OEIMs may be useful in drawing parallels with underlying disease activity. Previous studies have reported a higher prevalence of OEIMs in patients with increased IBD activity (8); however, other findings suggest that many OEIMs do not correlate directly with disease flares. (14) Thus, the evidence remains conflicting. In our cohort, a substantial proportion of patients with OEIMs exhibited a temporal association with active IBD, particularly in both CD and UC. (19) This observed relationship suggests that OEIMs may serve as valuable clinical markers of systemic disease activity, with their emergence or exacerbation potentially prompting earlier diagnostic evaluation or therapeutic escalation.

The management of OEIMs in IBD is inherently challenging due to their variable presentations, severity, and the absence of standardized treatment guidelines. As a result, therapeutic strategies are often empiric and focus on symptomatic relief, while also addressing the underlying gastrointestinal inflammation. OEIMs in our cohort frequently influenced IBD management decisions, leading to treatment escalation, including dose adjustments or the initiation of new therapies. These findings underscore the clinical relevance of OEIMs as not merely secondary phenomena but as markers of disease activity requiring active intervention.

Therapeutic approaches should be multifaceted. Systemic agents used in IBD, such as biologics, corticosteroids, azathioprine, methotrexate, dapsone, and colchicine, are often extended to OEIM treatment, with notable efficacy. (29) In our study, colchicine showed particular benefit in CD, while corticosteroids and topical tacrolimus were effective in UC. Given the adverse profile of long-term systemic corticosteroid use, corticosteroid-sparing agents are important considerations for chronic management.

Topical therapies remain central to symptomatic relief. Compounded or mixed mouthwashes containing anesthetics (e.g., viscous lidocaine) were the most commonly prescribed treatment in our cohort, highlighting the importance of local pain control. Topical corticosteroids are widely employed to manage early-stage lesions, such as cheilitis granulomatosa, mucosal tags, and aphthous ulcers, and may prevent progression or recurrence when combined with antimicrobial agents. Topical tacrolimus has demonstrated utility across a range of inflammatory oral dermatoses.(30) In our cohort, it was associated with improved results in CD. It can be applied as an ointment or compounded into a "swish-and-spit" solution for widespread mucosal involvement. For localized, refractory lesions, intralesional corticosteroids can be beneficial, although they were infrequently used in our population.

**Figure 1:**
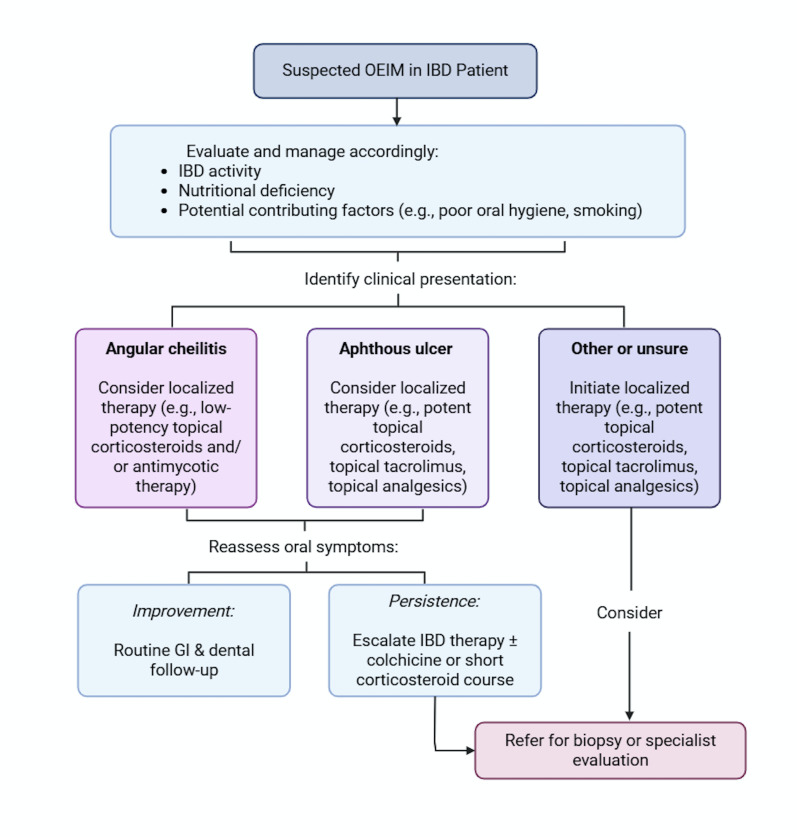
Proposed Algorithm for Management of OEIMs for Patients With IBD. GI indicates gastrointestinal; IBD, inflammatory bowel disease; OEIM, oral extraintestinal manifestation. (Used with permission of Mayo Foundation for Medical Education and Research, all rights reserved.) Created in BioRender. Aristizabal, M. (2025) https://BioRender.com/hcis82o.

Collectively, our findings emphasize the need for individualized, multidisciplinary care that integrates both systemic and local therapies. To aid clinical decision-making, we propose a diagnostic and treatment algorithm (Figure 1) to guide the evaluation and management of OEIMs in patients with IBD. Aphthous ulcers and angular cheilitis are presented as distinct diagnostic pathways because they have readily recognizable clinical features, allowing empiric treatment when examination findings are typical. In contrast, the "other or unsure" category includes a range of nonspecific oral diagnoses (e.g., unspecified stomatitis, unspecified mucositis) that are less distinctive on clinical exam and may require additional diagnostic evaluation before targeted therapy.

1

Limitations

This study has several limitations. Its retrospective design introduces potential bias, particularly in the selection of patients who may have presented with more severe or persistent oral symptoms. Biopsies were not uniformly performed, limiting histopathologic characterization. Furthermore, treatment responses were not standardized or systematically documented, making it difficult to assess the comparative efficacy of various interventions. Prospective studies are needed to further elucidate the pathogenesis of OEIMs, clarify their relationship with IBD activity, and evaluate the long-term outcomes of targeted therapies including colchicine and topical agents.

## Conclusions

OEIMs are a clinically significant yet often underrecognized component of IBD. In this multicenter retrospective study, they were prevalent in both CD and UC, frequently paralleling systemic disease activity and prompting therapeutic changes. These lesions add to the overall burden of IBD by exacerbating immune dysregulation, impairing nutrition, and adversely affecting quality of life and mental health.

Our findings support incorporating routine oral examinations into comprehensive IBD care. OEIMs should be viewed as potential early indicators of systemic disease activity or inadequate therapy, rather than incidental findings. Management should address lesion resolution and symptom relief while reassessing the adequacy of IBD treatment. Agents such as colchicine and topical tacrolimus may offer targeted benefit and merit further study.

The high prevalence of comorbidities in this cohort reinforces the need for multidisciplinary care addressing both gastrointestinal and extraintestinal manifestations. Patients with persistent or unclear oral lesions should be referred to an appropriate specialist for further evaluation, with biopsy considered when indicated and feasible. Enhanced recognition, timely diagnosis, and individualized management of OEIMs have the potential to improve disease control and patient outcomes.

## Figures and Tables

**Table 1:** Table Patient demographic characteristics.

Characteristic	Crohn's disease (n=67)	Ulcerative colitis (n=49)	Total (N=116)
Age, median (range), y	30.7 (18.1-77.8)	38.9 (19.6-74.7)	33.8 (18.1-77.8)
Gender			
Male	21 (31.3)	24 (49.0)	45 (38.8)
Female	46 (68.7)	25 (51.0)	71 (61.2)
Race			
Asian	1 (1.5)	0 (0)	1 (0.9)
Black	1 (1.5)	2 (4.1)	3 (2.6)
White	63 (94.0)	46 (93.9)	109 (94.0)
Other	2 (3.0)	1 (2.0)	3 (2.6)
Ethnicity			
Hispanic or Latino	4 (6.2)	1 (2.0)	5 (4.4)
Not Hispanic or Latino	61 (93.8)	48 (98.0)	109 (95.6)
Missing data	2	0	2

1

**Table 2:** Table Most common oral diagnoses given.

Oral Diagnosis	Crohn's Disease (N=67)	%	Ulcerative Colitis (N=49)	%
Aphthous ulcer	57	85.1%	36	73.5%
Stomatitis unspecified	8	11.9%	7	14.3%
Oral candidiasis	6	9.0%	3	6.1%
Mucositis unspecified	5	7.5%	6	12.2%
Angular cheilitis	4	6.0%	1	2.0%
Oral Crohn's disease	3	4.5%	0	0.0%
Glossitis	2	3.0%	0	0.0%
Mucogingivitis	2	3.0%	2	4.1%
Granulomatous cheilitis	1	1.5%	0	0.0%
Orofacial granulomatosis	1	1.5%	0	0.0%
Pyostomatitis vegetans	1	1.5%	1	2.0%
Cobblestone/hyperplastic oral mucosa	0	0.0%	1	2.0%

2

**Table 3:** Table Oral biopsy findings (n=25).

Biopsy finding	No. of patients (%)
Crohn's disease (n=14)	
Nonspecific findings	8 (57)
Oral Crohn's disease	3 (21)
Noncaseating granulomas	2 (14)
Lichenoid inflammation	1 (7)
Other granulomatous patterns	1 (7)
Other	3 (21)
Ulcerative colitis (n=11)	
Nonspecific findings	5 (45)
Lichenoid inflammation	4 (36)
Noncaseating granulomas	1 (9)
Other cheilitis	1 (9)
Other	1 (9)

3

**Table 4:** TableTreatments for oral findings (N=116).

Treatment	No. of patients (%)
Compounded or mixed mouthwash	60 (51.7)
Corticosteroid, topical	39 (33.6)
Corticosteroid, systemic	24 (20.7)
Anesthetic, topical	23 (19.8)
Antifungal, topical	17 (14.7)
Antifungal, systemic	9 (7.8)
Colchicine	8 (6.9)
Nutrition supplement	8 (6.9)
Tacrolimus, topical	6 (5.2)
Antiseptic mouthwash	4 (3.4)
Dapsone	4 (3.4)
Antibiotics, systemic (for oral disease)	3 (2.6)
Azathioprine	1 (0.9)
Infliximab (for oral disease)	1 (0.9)
Methotrexate	1 (0.9)
Corticosteroid, intralesional	1 (0.9)
Laser therapy	1 (0.9)

4

**Table 5:** Table OEIMs in IBD outcomes following treatment.

­Treatment for oral concerns	Improveda	Improved with IBD treatmentb	Required additional therapyc	Did not improve	Outcome not documented	Pvalue
Crohn's disease	(n=18)	(n=18)	(n=8)	(n=4)	(n=19)	
Colchicine	0 (0)	3 (17)	3 (38)	0 (0)	0 (0)	.009
Nutritional supplements	0 (0)	4 (22)	1 (13)	0 (0)	0 (0)	.05
Dapsone	0 (0)	2 (11)	2 (25)	0 (0)	0 (0)	.07
Tacrolimus, topical	3 (17)	0 (0)	1 (13)	0 (0)	0 (0)	.11
Corticosteroids, topical	7 (39)	9 (50)	6 (75)	0 (0)	7 (37)	.15
Other antibiotics, systemic (for oral disease)	0 (0)	0 (0)	1 (13)	0 (0)	0 (0)	.18
Antiseptic mouthwash	0 (0)	0 (0)	1 (13)	0 (0)	1 (5)	.33
Compounded or mixed mouthwash	10 (56)	11 (61)	5 (63)	2 (50)	8 (42)	.78
Anesthetics, topical	4 (22)	2 (11)	3 (38)	0 (0)	6 (32)	.39
Corticosteroids, systemic	3 (17)	4 (22)	1 (13)	2 (50)	5 (26)	.60
Azathioprine	0 (0)	1 (6)	0 (0)	0 (0)	0 (0)	.72
Methotrexate	0 (0)	1 (6)	0 (0)	0 (0)	0 (0)	.72
Infliximab (for oral disease)	0 (0)	1 (6)	0 (0)	0 (0)	0 (0)	.72
Antifungals, systemic	2 (11)	3 (17)	1 (13)	0 (0)	2 (11)	.97
Antifungals, topical	3 (17)	2 (11)	1 (13)	0 (0)	4 (21)	.98
Other	4 (22)	2 (11)	1 (13)	0 (0)	5 (26)	.73
Ulcerative colitis	(n=21)	(n=5)	(n=7)	(n=4)	(n=12)	
Corticosteroid, systemic	3 (14)	3 (60)	2 (29)	1 (25)	0 (0)	.03
Colchicine	0 (0)	0 (0)	2 (29)	0 (0)	0 (0)	.048
Tacrolimus, topical	0 (0)	0 (0)	2 (29)	0 (0)	0 (0)	.048
Corticosteroid, topical	2 (10)	0 (0)	3 (43)	2 (50)	3 (25)	.10
Compounded or mixed mouthwash	9 (43)	1 (20)	6 (86)	3 (75)	5 (42)	.15
Nutritional supplement	0 (0)	0 (0)	1 (14)	0 (0)	2 (17)	.24
Antifungal, topical	2 (10)	0 (0)	2 (29)	1 (25)	2 (17)	.48
Corticosteroid, intralesional	0 (0)	0 (0)	1 (14)	0 (0)	0 (0)	.33
Laser therapy	0 (0)	0 (0)	1 (14)	0 (0)	0 (0)	.33
Anesthetic, topical	3 (14)	0 (0)	1 (14)	1 (25)	3 (25)	.77
Antiseptic mouthwash	1 (5)	0 (0)	0 (0)	0 (0)	1 (8)	.99
Antifungal, systemic	1 (5)	0 (0)	0 (0)	0 (0)	0 (0)	.99
Other systemic antibiotics (for oral disease)	1 (5)	0 (0)	1 (14)	0 (0)	0 (0)	.61
Other	2 (10)	0 (0)	2 (29)	1 (25)	2 (17)	.48

IBD: inflammatory bowel disease. a. Improved without intervention or required only brief treatment (oral findings were not thought to be attributable to IBD).b. All patients required IBD treatment; some received additional treatment, as documented.c. Patients required additional therapy for persistent oral findings (no improvement with IBD treatment).

## Data Availability

Data will be made available on request, according to Mayo Clinic Foundation policies and regulations.
